# Human Milk Fatty Acid Composition of Allergic and Non-Allergic Mothers: The Ulm SPATZ Health Study

**DOI:** 10.3390/nu12061740

**Published:** 2020-06-10

**Authors:** Linda P. Siziba, Leonie Lorenz, Bernd Stahl, Marko Mank, Tamas Marosvölgyi, Tamas Decsi, Dietrich Rothenbacher, Jon Genuneit

**Affiliations:** 1 Pediatric Epidemiology, Department of Pediatrics, Medical Faculty, Leipzig University, 04103 Leipzig, Germany; Jon.Genuneit@medizin.uni-leipzig.de; 2 Institute of Epidemiology and Medical Biometry, Ulm University, 89075 Ulm, Germany; leonie.lorenz1@gmx.de (L.L.); Dietrich.Rothenbacher@uni-ulm.de (D.R.); 3 Danone Nutricia Research, 3584 CT Utrecht, The Netherlands; Bernd.Stahl@danone.com (B.S.); Marko.Mank@danone.com (M.M.); 4 Department of Chemical Biology & Drug Discovery, Utrecht Institute for Pharmaceutical Sciences, Utrecht University, 3584 CG Utrecht, The Netherlands; 5 Department of Paediatrics, Medical School, University of Pécs, H-7623 Pécs, Hungary; marosvolgyi.tamas@pte.hu (T.M.); decsi.tamas@pte.hu (T.D.)

**Keywords:** human milk, fatty acids, maternal allergy

## Abstract

The aim of this study was to determine the differences in human milk fatty acid composition in relation to maternal allergy within a large birth cohort study using statistical methods accounting for the correlations that exist in compositional data. We observed marginal differences in human milk fatty acid composition of allergic and non-allergic mothers. However, our results do not support the hypothesis that human milk fatty acid composition is influenced by allergy or that it differs between mothers with or without allergy. Observed differences in our results between transformed and untransformed fatty acid data call for re-evaluation of previous, as well as future, studies using statistical methods appropriate for compositionality of fatty acid data.

## 1. Introduction

Human milk contains a variety of nutrients, immunological and biological components necessary for growth and development of infants [1]. While human milk is considered the gold standard in early life nutrition, allergens, which may cause allergic reactions in atopic or allergic infants, can also be transferred through human milk [2]. Of great interest are fatty acids which have also been proposed to have immune-regulatory properties and could be involved in the etiology of allergic disease [3]. The essential fatty acids, linoleic acid (LA, C18:2n-6) and α-linoleic acid (ALA, C18:3n-3), are metabolized into long-chain polyunsaturated fatty acids (LCPUFAs) by the same set of desaturase and elongase enzymes [3].

In this regard, an impaired function of the delta-6-desaturase (D6D) enzyme converting LA and ALA to their long-chain metabolites has been implicated in atopic disease mechanisms. Several studies have found low concentrations of n-6 LCPUFAs in human milk of mothers with allergic disease [4,5,6], while other studies observed very little to no differences at all [1,7,8,9,10,11,12]. These studies compared human milk fatty acid composition of small subgroups, selected individuals or total fatty acids and did not account for existing correlations within compositional data. We, therefore, aimed at determining the differences in human milk fatty acid composition in relation to maternal allergy within a large birth cohort using statistical methods accounting for the constant sum constraint.

## 2. Materials and Methods

### 2.1. Study Design and Population

The Ulm SPATZ Health Study recruited a total of 1006 newborns and their 970 mothers (67% of all 1593 eligible families) from the general population during their hospital stay soon after delivery in the University Medical Center, Ulm, southern Germany, between April 2012 and May 2013. Details about the study can be found elsewhere [13]. Exclusion criteria were inadequate German language skills, outpatient childbirth, maternal age <18 years, postpartum transfer of mother or child to an intensive care unit, or stillbirth. Participation in the study was voluntary and informed consent was obtained from each study participant. Ethical approval was obtained from the Ethics Board of Ulm University (No. 311/11).

### 2.2. Data Collection and Measurements

Demographic information and maternal history of allergic disease (hay fever, asthma or atopic dermatitis) were collected using a self-administered questionnaire. Maternal allergy was self-reported and classified as mothers who reported a history of physician-diagnosed hay fever, neurodermatitis or asthma. Non-allergic mothers were classified as mothers who reported the absence of an allergic disease. Additional information was collected at approximately 6 weeks, 6 months and 12 months post-delivery through telephone interviews or postal self-administered questionnaires. The use of medication was assessed using an open questionnaire and the clear text entries were used to code and determine the use of allergy medication. Human milk samples were collected at approximately 6 weeks, 6 months and 12 months of lactation. Mothers were asked to manually express or pump breast milk between 9 am and 12 pm, after breakfast but before lunch and at least one hour after the last feed of the infant to reduce possible variations resulting from times of expression and feed. Human milk samples were stored at −80 °C until analysis of fatty acids between 2015 and 2018, as explained previously [13].

### 2.3. Statistical Analysis

Centered log ratio (CLR) transformations were applied to fatty acid data to account for compositionality [13]. Principal component analysis was used to evaluate associations of maternal allergy with possible fatty acid groups based on their correlational properties. The associations of maternal allergy with individual fatty acid concentrations were estimated using multiple linear regressions. Bonferroni adjustment was applied to account for multiple testing. Bonferroni correction dramatically reduces the probability of finding a significant result based on the number of tests performed. However, this also reduces the risk of making excessive assertions in clinical research [14]. All statistical analyses were performed with SAS version 9.4 (the SAS Institute, Cary, NC, USA).

## 3. Results

### 3.1. Characteristics of the Lactating Mothers

Of the 970 mothers enrolled into the SPATZ study, we included 475 lactating mothers with human milk samples available at both 6 weeks and 6 months. More than a third (36.2%, *n* = 175) reported a history of allergy. The lactating mothers were aged 33.5 ± 4.1 years and their characteristics are shown in Table 1. Of the lactating mothers who reported a history of allergy, *n* = 26 were on allergy medication. 

### 3.2. Fatty Acid Composition in Human Milk of Allergic and Non-Allegic Mothers 

The fatty acid composition of human milk samples collected at 6 weeks and 6 months was compared between allergic and non-allergic mothers. No significant differences were observed at 6 weeks of lactation. At 6 months, the CLR-transformed constituent sizes of the n-6 metabolites dihomo-γ-linolenic acid (DGLA, C20:3n-6; *p* = 0.0136), arachidonic acid (AA, C20:4n-6; *p* = 0.0359) and Osbond acid (C22:5n-6; *p* = 0.0134) were lower in mothers with allergic disease compared with their non-allergic counterparts (Table 2), but not statistically significant following Bonferroni correction (threshold α = 0.0009). Small differences were also observed when comparing untransformed relative proportions of fatty acids in human milk of allergic mothers to their non-allergic counterparts (Supplementary Table S1). Principal component analysis results did not show any clear associations of any maternal indicator with overall human milk fatty acid composition at any time point.

### 3.3. Associations between Human Milk Fatty Acid Composition and Maternal Allergy

Lactating mothers were stratified according to a specific allergic disease, i.e., asthma, hay fever or atopic dermatitis. We then investigated the associations between individual CLR-transformed fatty acids with asthma, hay fever and atopic dermatitis. Following adjustments for age, parity and pre-pregnancy BMI, the constituents of DGLA (β = 0.029, *p* = 0.0141) measured at 6 weeks were positively associated with maternal atopic dermatitis, while the constituents of DGLA (β = −0.015, *p* = 0.0105), AA (β = −0.003, *p* = 0.0186) and total n-6 LCPUFA (β = −0.043, *p* = 0.0125) measured at 6 months were negatively associated with maternal hay fever. In addition, at 6 weeks, the constituents of ALA (C18:3n-3; β = 0.176, *p* = 0.0065), dihomo-α-linolenic acid (C20:3n-3; β = 0.006, *p* = 0.0057) and total n-3 polyunsaturated fatty acids (PUFAs; β = 0.180, *p* = 0.0289) were positively associated with maternal asthma while LA (C18:2n-6; β = −0.011, *p* = 0.0481) was negatively associated with maternal asthma. D6D activity, calculated as the product/precursor ratio (18:2n-6/18:3n-6), was negatively associated with maternal asthma at both 6 weeks (β = −0.002, *p* = 0.0086) and 6 months (β = −0.002, *p* = 0.0079). However, these effect sizes were very small and all these associations lost significance following Bonferroni correction (threshold α = 0.0009).

## 4. Discussion

In the present study, we investigated the differences in human milk fatty acid composition of allergic and non-allergic mothers, sampled at both 6 weeks and 6 months. Our results suggest an impaired D6D enzyme activity converting LA to γ-linoleic acid (GLA) in allergic mothers. However, these associations were marginal and lost significance following correction for multiple testing. Therefore, these small differences could be driven by other factors that were not measured in this study.

Human milk fatty acid composition is driven by many factors and is also greatly influenced by gene variants of the fatty acid desaturase (*FADS*) gene cluster [15]. Lattka and colleagues [16] previously observed significant associations between the *FADS* genotype and AA contents in human milk as well as the ratio between AA and DGLA in the Ulm Birth Cohort Study (UBCS). Their results suggest a possible indication of the impact of *FADS* genotypes on the LCPUFA contents in human milk. In light of this, the UBCS and the Ulm SPATZ Health studies are similar with regards to both methodologies and population characteristics [17]. Therefore, we attribute these small differences that we observed to the genetic variation modulating the rate of endogenous synthesis on LCPUFAs. 

Nonetheless, we cannot rule out the potential influence of other biological human milk components as well as dietary intake. Although we did not assess dietary intake, the lactating mothers in this study were highly educated and presumably of higher social economic status, thus it is plausible that they were taking some LCPUFA supplements which could have contributed to the differences in fatty acid composition. In addition, previous reports [1,4,5,6,7,8,9,10,12,18] on differences in human milk fatty acid composition between non-allergic and allergic mothers show conflicting results. Similar to some studies [4,6,9,18], very small but significant differences were also observed when untransformed (relative proportions and/or absolute values of fatty acids) fatty acids were used to assess associations with asthma, hay fever and neurodermatitis. Thus, the observed differences in our results between CLR-transformed and -untransformed fatty acid data call for re-evaluation of previous studies using statistical methods appropriate for compositionality of fatty acid data. On one hand, even small differences in fatty acid composition of some lipid pools may have some physiological relevance in the etiology of disease and physiological aspects during infancy [3]. Although the influence of human milk fatty acid composition on child allergy has been studied extensively [19], whether the small differences observed in our study are associated with the development of an allergy in allergic infants of the respective mothers is yet to be studied. On the other hand, the mechanisms underlying the altered fatty acid compositions that exist in some groups of atopic individuals remain understudied. Therefore, unless these mechanisms are understood, it would be untimely to promote interventions that will influence fatty acid composition of allergic mothers as these may be ineffective [3].

The limitations of the study include the fact that we did not assess dietary intake during lactation as well as the lack of other parameters potentially relevant for this study as this may have influenced some of the fatty acid profiles. In addition, the number of lactating mothers on allergy medication was very low (*n* = 26) and we could not distinguish between active, chronic or acute allergy and whether or not the lactating mothers were in remission. Thus, granted that the timing of exposure is an important aspect which we could not measure in our study, our results should be interpreted with caution. Moreover, we cannot exclude the possibility of misclassification of the maternal history of allergy, as this was self-reported via questionnaires and included mothers with active and non-active allergic disease. 

## 5. Conclusions

In conclusion, our data do no support the hypothesis that human milk fatty acid composition is influenced by maternal allergy or that it differs between mothers with or without allergy. The small differences that we observed in this study could be ascribed to dietary habits, genetic factors and possibly other biological components in human milk. The conflicting results from previous studies also show the importance of studies that evaluate specific components in human milk and their biological role in infant and child health. Therefore, future studies including more homogenous groups of mothers with an active allergy are needed to investigate whether there are indeed differences and whether these differences have an impact on atopic sensitization in the infant.

## Figures and Tables

**Table 1:** Characteristics of allergic and non-allergic lactating women included in the Ulm SPATZ Health Study.

Characteristic	All Women (*n* = 475)		Allergy (*n* = 172)		No Allergy (*n* = 303)	
	*N*	% or Mean	*n*	% or Mean	*n*	% or Mean
Age	475	33.5	172	33.9	303	33.2
**Parity**						
0 births	245	51.6%	80	46.5%	165	54.5%
≥ 1 birth	230	48.4%	92	53.5%	138	45.5%
**Education**						
Low	14	3.0%	5	2.9%	9	3.0%
Intermediate	117	24.9%	37	21.8%	80	26.8%
High	338	72.1%	128	75.3%	210	70.2%
**Maternal BMI at 6 weeks**						
Underweight (BMI < 18.50)	3	0.7%	2	1.3%	1	0.4%
Normal (18.50 ≤ BMI < 25.00)	262	59.7%	88	55.7%	174	61.9%
Overweight (25.00 ≤ BMI < 30.00)	131	29.8%	47	29.7%	84	29.9%
Obese (BMI ≥ 30.00)	43	9.8%	21	13.3%	22	7.8%
**Maternal Allergy**						
Yes	172	36.2%	172	100.0%	303	100.0%
No	303	63.8%				
**Asthma**						
Yes	38	8.0%	38	22.1%		
No	437	92.0%	134	77.9%	303	100.0%
**Hay fever**						
Yes	120	25.3%	120	69.8%		
No	355	74.7%	52	30.2%	303	100.0%
**Neurodermatitis**						
Yes	67	14.1%	67	39.0%		
No	408	85.9%	105	61.0%	303	100.0%

BMI—body mass index. Sums may not always add up to total because certain items were missing. Percentages exclude those missing items.

**Table 2:** Means and standard deviations (mean (SD)) of centered log ratio (CLR)-transformed fatty acid concentrations of human milk samples measured at 6 weeks and 6 months of lactation.

Fatty Acid		All 6 Weeks Samples (*n* = 475)		Allergy (*n* = 172)		No Allergy (*n* = 303)		*p*	All 6 Months Samples (*n* = 475)		Allergy (*n* = 172)		No Allergy (*n* = 303)		*p*
SFAs		3.47	(0.13)	3.48	(0.15)	1.16	(0.12)	0.9853	3.58	(0.13)	3.59	(0.14)	3.58	(0.13)	0.7641
C8:0	Caprylic	0.19	(0.52)	0.17	(0.68)	0.20	(0.41)	0.9653	0.28	(0.36)	0.29	(0.34)	0.28	(0.37)	0.9731
C10:0	Capric	2.25	(0.30)	2.24	(0.32)	2.25	(0.29)	0.7934	2.33	(0.29)	2.32	(0.29)	2.33	(0.28)	0.7049
C11:0	Undecylic	−2.32	(0.31)	−2.34	(0.30)	−2.31	(0.31)	0.3613	−2.27	(0.29)	−2.27	(0.30)	−2.27	(0.29)	0.8034
C12:0	Lauric	3.39	(0.34)	3.39	(0.37)	3.39	(0.33)	0.8125	3.61	(0.31)	3.60	(0.32)	3.62	(0.30)	0.4720
C13:0	Tridecylic	−1.58	(0.24)	−1.59	(0.23)	−1.58	(0.24)	0.5101	−1.47	(0.28)	−1.46	(0.24)	−1.48	(0.30)	0.9892
C14:0	Myristic	3.54	(0.22)	3.53	(0.24)	3.55	(0.22)	0.3139	3.77	(0.23)	3.77	(0.23)	3.78	(0.23)	0.6136
C15:0	Pentadecylic	0.81	(0.23)	0.80	(0.23)	0.81	(0.24)	0.5808	0.86	(0.23)	0.85	(0.23)	0.87	(0.23)	0.3542
C16:0	Palmitic	4.88	(0.15)	4.89	(0.16)	4.88	(0.14)	0.2241	4.93	(0.14)	4.93	(0.13)	4.93	(0.14)	0.5410
C17:0	Margaric	0.59	(0.17)	0.59	(0.17)	0.60	(0.17)	0.4968	0.66	(0.15)	0.66	(0.15)	0.66	(0.16)	0.8611
C18:0	Stearic	3.68	(0.23)	3.70	(0.27)	3.67	(0.21)	0.2180	3.76	(0.22)	3.78	(0.22)	3.75	(0.22)	0.0409
C19:0	Nonadecylic	−1.85	(0.24)	−1.83	(0.24)	−1.85	(0.24)	0.2386	−1.79	(0.35)	−1.78	(0.43)	−1.80	(0.29)	0.2133
C20:0	Arachidic	0.15	(0.24)	0.18	(0.28)	0.14	(0.22)	0.0958	0.20	(0.23)	0.23	(0.24)	0.18	(0.23)	0.0370
C22:0	Behenic	−0.72	(0.24)	−0.71	(0.25)	−0.72	(0.24)	0.6782	−0.67	(0.26)	−0.65	(0.29)	−0.67	(0.25)	0.8018
C23:0	Tricosylic	−4.29	(2.10)	−4.36	(2.12)	−4.24	(2.09)	0.7713	−4.08	(2.06)	−4.04	(2.05)	−4.10	(2.07)	0.4891
C24:0	Lignoceric	−1.14	(0.37)	−1.13	(0.35)	−1.15	(0.38)	0.5647	−1.20	(0.44)	−1.17	(0.42)	−1.22	(0.44)	0.5063
MUFAs		3.36	(0.15)	3.36	(0.15)	−1.16	(0.12)	0.5856	3.40	(0.13)	3.41	(0.13)	3.40	(0.13)	0.8740
C12:1n-1		−2.12	(0.38)	−2.12	(0.35)	−2.11	(0.39)	0.5121	−2.08	(0.37)	−2.07	(0.34)	−2.09	(0.39)	0.9324
C14:1n-5	Myristoleic	0.53	(0.27)	0.52	(0.26	0.54	(0.27)	0.2286	0.57	(0.28)	0.57	(0.27)	0.57	(0.28)	0.7140
C16:1n-7	Palmitoleic	2.64	(0.29)	2.64	(0.29)	2.63	(0.30)	0.9015	2.60	(0.28)	2.59	(0.27)	2.61	(0.28)	0.4285
C18:1n-9	Vaccenic	5.30	(0.19)	5.29	(0.21	5.30	(0.18)	0.8530	5.34	(0.17)	5.34	(0.17)	5.34	(0.17)	0.9269
C18:1n-7	Oleic	2.27	(0.22)	2.27	(0.22)	2.27	(0.21)	0.6890	2.24	(0.25)	2.24	(0.21)	2.24	(0.27)	0.6195
C20:1n-9	Eicosenoic	0.90	(0.21)	0.90	(0.22)	0.89	(0.21)	0.7753	0.83	(0.23)	0.84	(0.24)	0.83	(0.22)	0.5270
C22:1n-9	Erucic	−0.83	(0.25)	−0.83	(0.26)	−0.83	(0.24)	0.7583	−0.92	(0.28)	−0.91	(0.29)	−0.93	(0.27)	0.2574
C24:1n-9	Nervonic	−1.16	(0.59)	−1.17	(0.61)	−1.16	(0.58)	0.9745	−1.26	(0.52)	−1.26	(0.52)	−1.26	(0.51)	0.7422
Trans-FAs		−1.13	(0.57)	−1.11	(0.53)	3.47	(0.13)	0.7707	−1.02	(0.56)	−0.98	(0.58)	−1.04	(0.54)	0.8431
C14:1n-5t	Myristelaidic	−4.24	(0.58)	−4.27	(0.62)	−4.22	(0.55)	0.3249	−4.20	(0.57)	−4.19	(0.53)	−4.21	(0.59)	0.9244
C15:1n-5t		−4.16	(0.50)	−4.15	(0.39)	−4.17	(0.55)	0.7865	−4.15	(0.50)	−4.16	(0.48)	−4.15	(0.52)	0.8666
C16:1n-7t		−1.97	(0.56)	−1.99	(0.62)	−1.96	(0.52)	0.9266	−1.98	(0.56)	−1.97	(0.56)	−1.98	(0.57)	0.7485
T18:1		0.72	(0.88)	0.74	(0.84)	0.70	(0.91)	0.6301	0.84	(0.81)	0.88	(0.78)	0.82	(0.83)	0.4712
C18:2n6-tt	Linolelaidic	−2.37	(1.51)	−2.28	(1.45)	−2.42	(1.55)	0.5086	−2.23	(1.50)	−2.12	(1.39)	−2.29	(1.56)	0.4403
BCFAs		−1.81	(0.34)	−1.83	(0.34)	2.09	(0.24)	0.6450	−1.72	(0.33)	−1.72	(0.32)	−1.72	(0.33)	0.3358
C15ai	Anteisopentadecylic	−0.43	(0.37)	−0.45	(0.33)	−0.41	(0.38)	0.3447	−0.34	(0.35)	−0.34	(0.35)	−0.34	(0.36)	0.7713
C16i	Isopalmitic	−0.70	(0.28)	−0.72	(0.27)	−0.70	(0.28)	0.4236	−0.64	(0.33)	−0.63	(0.27)	−0.64	(0.36)	0.7147
C18i	Anteisopentadecylic	−1.89	(0.24)	−1.92	(0.25)	−1.88	(0.24)	0.0791	−1.83	(0.23)	−1.84	(0.23)	−1.82	(0.22)	0.3359
PUFAs		2.21	(0.23)	2.20	(0.24)	3.36	(0.15)	0.3504	2.26	(0.22)	2.24	(0.20)	2.27	(0.23)	0.8778
C18:2n6	Linoleic	4.03	(0.29)	4.01	(0.32)	4.04	(0.28)	0.2269	4.09	(0.28)	4.06	(0.25)	4.11	(0.29)	0.1221
C18:3n6	γ-linolenic	−0.41	(0.36)	−0.42	(0.41)	−0.41	(0.33)	0.9401	−0.56	(0.35)	−0.59	(0.36)	−0.54	(0.34)	0.0686
C20:2n-6	Eicosadienoic	0.40	(0.24)	0.38	(0.24)	0.41	(0.23)	0.2493	0.25	(0.24)	0.23	(0.23)	0.26	(0.24)	0.1938
C20:3n-6	Dihomo-γ-linolenic	0.68	(0.25)	0.68	(0.26)	0.68	(0.25)	0.9997	0.41	(0.24)	0.38	(0.23)	0.43	(0.24)	0.0136
C20:4n-6	Arachidonic	0.86	(0.22)	0.87	(0.24)	0.86	(0.22)	0.6266	0.82	(0.23)	0.80	(0.23)	0.84	(0.22)	0.0359
C22:2n-6	Docosadienoic	−1.95	(0.62)	−1.91	(0.36)	−1.97	(0.73)	0.8907	−2.32	(1.00)	−2.33	(0.95)	−2.32	(1.03)	0.4210
C22:4n-6	Adrenic	−0.79	(0.36)	−0.77	(0.28)	−0.81	(0.40)	0.1586	−0.84	(0.34)	−0.86	(0.44)	−0.82	(0.26)	0.4940
C22:5n-6	Osbond	−1.53	(0.36)	−1.51	(0.31)	−1.54	(0.38)	0.3628	−1.67	(0.38)	−1.72	(0.38)	−1.64	(0.38)	0.0134
Σn-6 PUFA		2.08	(0.25)	2.06	(0.26)	2.07	(0.25)	0.4362	2.13	(0.24)	2.11	(0.21)	2.14	(0.25)	0.1709
Σn-6 LCPUFA		−0.17	(0.16)	−0.17	(0.16)	−0.85	(0.24)	0.9706	−0.30	(0.15)	−0.31	(0.15)	−0.29	(0.16)	0.3544
C18:3n-3	α-linoleic	1.60	(0.38)	1.59	(0.41)	1.61	(0.37)	0.3960	1.69	(0.38)	1.69	(0.37)	1.69	(0.39)	0.6836
C20:3n-3	Dihomo-α-linoleic	−1.44	(0.36)	−1.44	(0.31)	−1.44	(0.39)	0.4449	−1.59	(0.47)	−1.59	(0.52)	−1.59	(0.45)	0.7333
C20:4n-3		−0.71	(0.32)	−0.70	(0.32)	−0.71	(0.32)	0.7514	−1.05	(0.32)	−1.07	(0.33)	−1.04	(0.31)	0.5243
C20:5n-3	Eicosapentaenoic	−0.98	(0.36)	−0.95	(0.37)	−0.99	(0.36)	0.3504	−1.01	(0.39)	−1.02	(0.39)	−1.01	(0.39)	0.8069
C22:5n-3	Docosapentanoic	−0.18	(0.22)	−0.17	(0.23)	−0.19	(0.22)	0.6234	−0.15	(0.22)	−0.16	(0.23)	−0.14	(0.21)	0.2787
C22:6n-3	Docosahexaenoic	0.35	(0.39)	0.34	(0.42)	0.35	(0.38)	0.9592	0.20	(0.44)	0.18	(0.44)	0.21	(0.44)	0.8271
Σn-3 PUFAs		0.07	(0.27)	0.07	(0.29)	−1.14	(0.58)	0.9518	0.09	(0.28)	0.10	(0.27)	0.09	(0.29)	0.4032
Σn-3 LCPUFAs		−0.84	(0.25)	−0.84	(0.25)	2.22	(0.22)	0.9518	−0.94	(0.27)	−0.95	(0.28)	−0.94	(0.26)	0.4032
Met Index	Metabolic Index	1.16	(0.14)	1.16	(0.16)	−0.17	(0.16)	0.2407	1.16	(0.14)	1.17	(0.15)	1.16	(0.13)	0.2585
D6D	D6D activity	−1.16	(0.14)	−1.16	(0.16)	−1.79	(0.35)	0.5772	−1.16	(0.14)	−1.17	(0.15)	−1.16	(0.13)	0.6815

FA—fatty acid; SFAs—saturated fatty acids; MUFAs—monounsaturated fatty acids; BCFAs—branched chain fatty acids; PUFAs—polyunsaturated fatty acids; D6D—delta-6-desaturase. *p*—*p* values derived from the Wilcoxon signed-rank test comparing fatty acid concentrations between allergic and non-allergic groups at each time point. Bonferroni-adjusted level of statistical significance is α = 0.05/56 = 0.0009.

## Supplementary Materials

The following are available online at https://www.mdpi.com/2072-6643/12/6/1740/s1, Table S1: Means and standard deviations (mean (SD)) of relative proportions (%) of total fatty acid concentrations in human milk sampled at 6 weeks and 6 months of lactation in the Ulm SPATZ Health Study.
