# Supplementary material for: Human Milk Fatty Acid Composition of Allergic and Non-Allergic Mothers: The Ulm SPATZ Health Study
Source: Nutrients. 2020 Jun 10;12(6):1740. doi: 10.3390/nu12061740 (PMC7352579; doi:10.3390/nu12061740)
Supplement: Supplementary file 1 [file nutrients-12-01740-s001.pdf]

Table S1. Means and standard deviations (Mean (SD)) of relative proportions (%) of total fatty acid concentrations in human milk sampled at 6 weeks and 6 months of lactation in The Ulm SPATZ Health Study.

Fatty Acid		All 6 weeks samples (*n* = 475)		Allergy (*n* = 172)		No Allergy (*n* = 303)		*p* value	All 6 months samples (*n* = 475)		Allergy (*n* = 172)		No Allergy (*n* = 303)		*p* value
SFA		45.52	(4.58)	45.80	(4.97)	0.12	(0.03)	0.1796	47.09	(4.32)	47.37	(4.22)	46.93	(4.37)	0.2133
C8:0	Caprylic	0.23	(0.08)	0.23	(0.08)	0.23	(0.08)	0.8467	0.23	(0.08)	0.23	(0.07)	0.23	(0.08)	0.7503
C10:0	Capric	1.74	(0.50)	1.74	(0.54)	1.74	(0.48)	0.8166	1.75	(0.45)	1.75	(0.45)	1.75	(0.45)	0.9861
C11:0	Undecylic	0.02	(0.01)	0.02	(0.01)	0.02	(0.01)	0.3535	0.02	(0.01)	0.02	(0.01)	0.02	(0.01)	0.8453
C12:0	Lauric	5.53	(1.93)	5.55	(2.08)	5.52	(1.85)	0.8811	6.38	(1.80)	6.32	(1.77)	6.42	(1.81)	0.7681
C13:0	Tridecylic	0.04	(0.01)	0.04	(0.01)	0.04	(0.01)	0.5268	0.04	(0.01)	0.04	(0.01)	0.04	(0.01)	0.9822
C14:0	Myristic	6.29	(1.62)	6.22	(1.65)	6.34	(1.60)	0.4138	7.40	(1.78)	7.36	(1.70)	7.42	(1.83)	0.8633
C15:0	Pentadecylic	0.41	(0.12)	0.41	(0.12)	0.41	(0.12)	0.5820	0.41	(0.11)	0.41	(0.12)	0.41	(0.11)	0.4633
C16:0	Palmitic	23.39	(2.41)	23.55	(2.58)	23.29	(2.30)	0.1269	22.92	(2.38)	23.09	(2.30)	22.82	(2.42)	0.2588
C17:0	Margaric	0.33	(0.07)	0.32	(0.06)	0.33	(0.07)	0.4376	0.32	(0.06)	0.33	(0.06)	0.32	(0.06)	0.7153
C18:0	Stearic	7.15	(1.41)	7.32	(1.66)	7.05	(1.24)	0.1958	7.23	(1.33)	7.43	(1.34)	7.12	(1.32)	0.0251
C19:0	Nonadecylic	0.03	(0.01)	0.03	(0.01)	0.03	(0.01)	0.3644	0.03	(0.01)	0.03	(0.01)	0.03	(0.01)	0.2101
C20:0	Arachidic	0.21	(0.04)	0.22	(0.05)	0.21	(0.04)	0.0862	0.21	(0.04)	0.21	(0.04)	0.20	(0.04)	0.0101
C22:0	Behenic	0.09	(0.02)	0.09	(0.02)	0.09	(0.02)	0.8863	0.09	(0.02)	0.09	(0.03)	0.09	(0.02)	0.3737
C23:0	Tricosylic	0.01	(0.01)	0.01	(0.01)	0.01	(0.01)	0.6803	0.01	(0.01)	0.01	(0.01)	0.01	(0.01)	0.4710
C24:0	Lignoceric	0.06	(0.02)	0.06	(0.02)	0.06	(0.02)	0.7356	0.05	(0.02)	0.06	(0.03)	0.05	(0.02)	0.3841
MUFA		40.57	(3.57)	40.43	(3.67)	0.01	(0.00)	0.3998	39.37	(3.30)	39.42	(3.16)	39.34	(3.38)	0.9498
C12:1n-1		0.02	(0.01)	0.02	(0.01)	0.02	(0.01)	0.5507	0.02	(0.01)	0.02	(0.01)	0.02	(0.01)	0.9437
C14:1n-5	Myristoleic	0.32	(0.10)	0.31	(0.10)	0.32	(0.10)	0.3033	0.31	(0.10)	0.31	(0.10)	0.31	(0.09)	0.6836
C16:1n-7	Palmitoleic	2.56	(0.73)	2.56	(0.76)	2.56	(0.71)	0.9329	2.31	(0.65)	2.29	(0.66)	2.33	(0.64)	0.4323
C18:1n-9	Vaccenic	35.36	(3.43)	35.22	(3.63)	35.44	(3.31)	0.4783	34.64	(3.14)	34.69	(3.05)	34.61	(3.19)	0.8831
C18:1n-7	Oleic	1.73	(0.31)	1.73	(0.34)	1.73	(0.29)	0.7223	1.58	(0.28)	1.59	(0.29)	1.58	(0.28)	0.9233
C20:1n-9	Eicosenoic	0.44	(0.08)	0.44	(0.08)	0.44	(0.08)	0.6380	0.39	(0.09)	0.39	(0.09)	0.39	(0.09)	0.4513
C22:1n-9	Erucic	0.08	(0.02)	0.08	(0.02)	0.08	(0.02)	0.9504	0.07	(0.02)	0.07	(0.02)	0.07	(0.02)	0.2183
C24:1n-9	Nervonic	0.06	(0.02)	0.06	(0.02)	0.06	(0.02)	0.9698	0.05	(0.02)	0.05	(0.02)	0.05	(0.02)	0.8932
Trans-FA		0.54	(0.34)	0.55	(0.37)	45.36	(4.35)	0.6237	0.56	(0.41)	0.60	(0.51)	0.55	(0.34)	0.4807
C14:1n-5t	Myristelaidic	0.00	(0.00)	0.00	(0.00)	0.00	(0.00)	0.4036	0.00	(0.00)	0.00	(0.00)	0.00	(0.00)	0.9681
C15:1n-5t		0.00	(0.00)	0.00	(0.00)	0.00	(0.00)	0.7751	0.00	(0.00)	0.00	(0.00)	0.00	(0.00)	0.8608
C16:1n-7t		0.03	(0.01)	0.03	(0.01)	0.03	(0.01)	0.9252	0.03	(0.01)	0.03	(0.01)	0.03	(0.01)	0.8085
T18:1		0.46	(0.32)	0.47	(0.35)	0.46	(0.31)	0.6749	0.49	(0.39)	0.52	(0.49)	0.47	(0.32)	0.4220
C18:2n6-tt	Linolelaidic	0.04	(0.05)	0.04	(0.05)	0.04	(0.05)	0.5339	0.04	(0.05)	0.05	(0.05)	0.04	(0.05)	0.4170
BCFAs		0.25	(0.11)	0.24	(0.08)	11.64	(2.56)	0.3467	0.25	(0.08)	0.25	(0.08)	0.25	(0.08)	0.7636
C15ai	Anteisopentadecylic	0.13	(0.09)	0.12	(0.05)	0.13	(0.10)	0.3589	0.13	(0.05)	0.13	(0.05)	0.13	(0.05)	0.8304
C16i	Isopalmitic	0.09	(0.03)	0.09	(0.03)	0.09	(0.03)	0.4975	0.09	(0.03)	0.09	(0.03)	0.09	(0.03)	0.7707
C18i	Anteisopentadecylic	0.03	(0.01)	0.03	(0.01)	0.03	(0.01)	0.2101	0.03	(0.01)	0.03	(0.01)	0.03	(0.01)	0.6742
PUFA		13.13	(2.87)	12.98	(3.14)	40.65	(3.51)	0.2393	12.73	(2.86)	12.37	(2.43)	12.93	(3.06)	0.0870
C18:2n6	Linoleic	10.23	(2.58)	10.08	(2.81)	10.31	(2.44)	0.1851	10.14	(2.59)	9.82	(2.19)	10.33	(2.78)	0.0888
C18:3n6	γ-linolenic	0.12	(0.04)	0.12	(0.05)	0.12	(0.04)	0.0373	0.10	(0.03)	0.10	(0.04)	0.10	(0.03)	0.0720
C20:2n-6	Eicosadienoic	0.27	(0.05)	0.26	(0.05)	0.27	(0.06)	0.2024	0.22	(0.04)	0.21	(0.04)	0.22	(0.04)	0.1052
C20:3n-6	Dihomo-γ-linolenic	0.36	(0.09)	0.36	(0.10)	0.36	(0.08)	0.5911	0.26	(0.06)	0.25	(0.05)	0.26	(0.06)	0.0172
C20:4n-6	Arachidonic	0.43	(0.08)	0.43	(0.09)	0.43	(0.08)	0.8152	0.38	(0.08)	0.38	(0.08)	0.39	(0.08)	0.0821
C22:2n-6	Docosadienoic	0.03	(0.01)	0.03	(0.01)	0.03	(0.01)	0.9628	0.02	(0.01)	0.02	(0.01)	0.02	(0.01)	0.4248
C22:4n-6	Adrenic	0.08	(0.02)	0.08	(0.02)	0.08	(0.02)	0.3324	0.07	(0.02)	0.07	(0.02)	0.07	(0.02)	0.8133
C22:5n-6	Osbond	0.04	(0.02)	0.04	(0.01)	0.04	(0.02)	0.5894	0.03	(0.01)	0.03	(0.01)	0.03	(0.01)	0.0231
	Σn-6 PUFA	11.55	(2.71)	1.40	(2.95)	11.57	(0.46)	0.0331	11.23	(2.67)	10.88	(2.26)	11.43	(2.87)	0.0689
	Σn-6 LCPUFA	1.21	(0.21)	1.20	(0.23)	0.63	(0.22)	0.6412	0.98	(0.16)	0.96	(0.15)	1.00	(0.17)	0.0332
C18:3n3	α -linoleic	0.94	(0.38)	0.94	(0.42)	0.94	(0.36)	0.5092	0.97	(0.41)	0.96	(0.38)	0.97	(0.42)	0.5419
C20:3n-3	Dihomo-α-linoleic	0.04	(0.01)	0.04	(0.01)	0.04	(0.01)	0.5647	0.04	(0.01)	0.04	(0.01)	0.04	(0.01)	0.7093
C20:4n-3		0.09	(0.04)	0.09	(0.04)	0.09	(0.04)	0.9986	0.06	(0.02)	0.06	(0.02)	0.06	(0.02)	0.6131
C20:5n-3	Eicosapentaenoic	0.07	(0.04)	0.07	(0.04)	0.07	(0.03)	0.5595	0.07	(0.04)	0.07	(0.03)	0.07	(0.04)	0.9598
C22:5n-3	Docosapentanoic	0.15	(0.04)	0.15	(0.04)	0.15	(0.04)	0.6931	0.15	(0.04)	0.15	(0.04)	0.15	(0.04)	0.5090
C22:6n-3	Docosahexaenoic	0.27	(0.13)	0.27	(0.13)	0.27	(0.13)	0.8380	0.23	(0.12)	0.22	(0.12)	0.23	(0.13)	0.9114
	Σn-3 PUFA	1.58	(0.49)	1.58	(0.53)	0.53	(0.33)	0.6151	1.50	(0.50)	1.49	(0.47)	1.51	(0.52)	0.9700
	Σn-3 LCPUFA	0.63	(0.22)	0.64	(0.23)	13.21	(2.71)	0.8179	0.54	(0.20)	0.53	(0.19)	0.54	(0.20)	0.4738
Met Index	Metabolic Index	0.12	(0.03)	0.13	(0.03)	1.21	(0.20)	0.3084	0.10	(0.02)	0.10	(0.02)	0.10	(0.02)	0.7875
d6d	D6D activity	0.01	(0.00)	0.01	(0.00)	0.25	(0.13)	0.4200	0.01	(0.00)	0.01	(0.00)	0.01	(0.00)	0.4968

SFA- Saturated fatty acids; MUFA- Monounsaturated fatty acids; BCFA- Branched chain fatty acids; PUFA- Polyunsaturated fatty acids. P values derived from Wilcoxon signed-rank test comparing fatty acid concentrations between allergic and non-allergic groups at each time point. Bonferroni-adjusted level of statistical significance is α=0.05/57 = 0.0009.
